# Vitamin D Enhances Anticancer Properties of Cediranib, a VEGFR Inhibitor, by Modulation of VEGFR2 Expression in Melanoma Cells

**DOI:** 10.3389/fonc.2021.763895

**Published:** 2021-12-24

**Authors:** Anna Piotrowska, Fernando Pereira Beserra, Justyna Marta Wierzbicka, Joanna Irena Nowak, Michał Aleksander Żmijewski

**Affiliations:** ^1^ Department of Histology, Faculty of Medicine, Medical University of Gdańsk, Gdańsk, Poland; ^2^ Institute of Biosciences, São Paulo State University, São Paulo, Brazil

**Keywords:** melanoma, vitamin D, calcipotriol, cediranib, anti-angiogenic therapy, VEGFR, VEGFR inhibitors

## Abstract

Regardless of the recent groundbreaking introduction of personalized therapy, melanoma continues to be one of the most lethal skin malignancies. Still, a substantial proportion of patients either fail to respond to the therapy or will relapse over time, representing a challenging clinical problem. Recently, we have shown that vitamin D enhances the effectiveness of classical chemotherapeutics in the human malignant melanoma A375 cell line. In search for new combination strategies and adjuvant settings to improve melanoma patient outcomes in the current study, the effects of cediranib (AZD2171), an oral tyrosine kinase inhibitor of VEGFR1-3, PDGFR, and c-KIT, used in combination either with 1,25(OH)_2_D_3_ or with low-calcemic analog calcipotriol were tested on four human malignant melanoma cell lines (A375, MNT-1, RPMI-7951, and SK-MEL-28). Melanoma cells were pretreated with vitamin D and subsequently exposed to cediranib. We observed a marked decrease in melanoma cell proliferation (A375 and SK-MEL-28), G2/M cell cycle arrest, and a significant decrease in melanoma cell mobility in experimental conditions used (A375). Surprisingly, concurrently with a very desirable decrease in melanoma cell proliferation and mobility, we noticed the upregulation of VEGFR2 at both protein and mRNA levels. No effect of vitamin D was observed in MNT-1 and RPMI-7951 melanoma cells. It seems that vitamin D derivatives enhance cediranib efficacy by modulation of VEGFR2 expression in melanoma cells expressing VEGFR2. In conclusion, our experiments demonstrated that vitamin D derivatives hold promise as novel adjuvant candidates to conquer melanoma, especially in patients suffering from vitamin D deficiency. However, further extensive research is indispensable to reliably assess their potential benefits for melanoma patients.

## Introduction

Melanoma, although representing a minor fraction of all skin malignancies, remains the most lethal form (1, 2). Before the modern era, patients with advanced melanoma could expect a 5-year survival rate of 10% (3). Beginning in 2011, novel therapies, including immunotherapy with immune checkpoint inhibitors CTLA-4 or PD-1, as well as targeted therapy with BRAF and MEK inhibitors, have become a key breakthrough in the clinical landscape of melanoma treatment (4). Unprecedented in cancer medicine, from 2013 to 2016, overall melanoma mortality decreased by 17.9% (5). Nevertheless, regardless of the groundbreaking treatment options, most patients invariably relapse from BRAF/MEK signaling inhibition within a year from treatment commencement (6). About 50% of patients treated with immune checkpoints inhibitors do not respond due to primary resistance and a great proportion of responders experience tumor relapse within 2 years (7, 8). Current 5-year survival rate for metastatic melanoma is therefore 27% (9). What is more, the incidence of melanoma is constantly rising worldwide, and currently, melanoma is expected to be the fifth most common cancer in both males and females, as estimated by the American Cancer Society (9). Therefore, it seems to be highly reasonable to focus on new combination strategies and adjuvant settings to improve melanoma patient outcomes (4).

Vitamin D is a secosteroid endogenously produced in the skin from its precursor, 7-dehydrocholesterol, using the energy of UVB irradiation (10, 11). It should be emphasized that vitamin D3 is biologically inert and requires two subsequent hydroxylations to gain its hormonal functions. First, hydroxylation at C-25 takes place in the liver, and second, at C-1α in kidneys, giving the most active form 1,25(OH)_2_D_3_, calcitriol (11, 12). The extrarenal expression of vitamin D hydroxylases was proven in many different sites, such as lymph nodes, placenta, breast, and colon (13); however, it should be underlined that the skin is the only organ equipped with the whole pathway of vitamin D synthesis and activation (14, 15). Apart from a historically known role in regulation of calcium homeostasis, vitamin D has widely appreciated anticancer properties, including antiproliferative, antiangiogenic, and pro-differentiative effects in various types of cancer (10). Therefore, vitamin D is considered for cancer prevention, as the recent VITAL study (16–18) and some former studies (19, 20) suggest that vitamin D supplementation has beneficial effects in reducing risks of cancer. A very recent study has shown that among patients with newly resected stage II melanoma who received adjuvant vitamin D3 (100,000 IU every 50 days), individuals with low Breslow score (<3 mm) had a double increase in 25OHD levels from baseline after 4 months, whereas patients with Breslow score ≥3 mm had a significantly lower increase over time. After 12 months, subjects with low 25OHD levels and Breslow score ≥3 mm had shorter disease-free survival (*p* = 0.02) compared to those with Breslow score <3 mm and/or high levels of 25OHD (21). At baseline, 80% of these melanoma patients were vitamin D insufficient (21). This observation underlines the role of vitamin D supplementation status of patients in melanoma prognosis. Indeed, the National Institute for Health and Care Excellence guidelines clearly recommend that assessment of vitamin D levels and relevant advice should be an inherent aspect of the management of patients with melanoma at the secondary care level (22). Currently, the role of vitamin D supplementation on cutaneous malignant melanoma outcome is assessed in the ViDMe trial (23). Additionally, an inverse correlation has also been documented between the expression of the vitamin D receptor (VDR) and a crucial vitamin D activating enzyme (CYP27B1) with melanoma progression and disease outcome (24–26). Furthermore, as revealed by analysis of transcriptome of melanoma patients, VDR expression was independently protective for melanoma-related death in both primary and metastatic disease (27). What is more, it was shown that active forms of vitamin D improve efficacy of several anticancer drugs, such as cisplatin (28, 29), dacarbazine (30), doxorubicin (31), and proton therapy (32). It is also suggested that vitamin D immune-modulating ability could offer indications for a novel vitamin D application in melanoma patients receiving immunotherapy (33).

Currently, the upper normal limit of 25(OH)D in blood serum, used in clinic as a biomarker of vitamin D status (34), is defined at 100 ng/ml (35). A recent study suggests that extended intakes of 20,000 IU/day to 60,000 IU/day, associated with 25OHD blood levels ranging as high as 384 mg/dl, were found to be safe without any evidence of toxicity (36). However, considering patient safety, the major disadvantage of vitamin D and its natural active metabolite—1,25(OH)_2_D_3_—is that prolonged supplementation with high doses (>50,000 IU per day for several months), which could be beneficial in the cancer therapy, may also lead, although not necessarily, to hypercalcemia (12, 37). In our constant work to select most potent but low calcemic vitamin D analogs, we have investigated the series of CYP11A1 metabolites of vitamin D (30, 38, 39), which are products of a recently discovered novel pathway of vitamin D metabolism and activation (40–43), modified vitamin D_2_ analogs (44), and vitamin D analogs with the shortened side chain (15, 45) as to their efficacy against melanoma cell lines. Simultaneously, we have also explored whether vitamin D and its non- or low-calcemic analogs will enhance the effectiveness of classical chemotherapeutics, cisplatin and dacarbazine, in the human malignant melanoma A375 cell line (30). We showed that both calcitriol and calcipotriol exhibited modulatory effects on the melanoma cells treated with dacarbazine, decreasing the half maximal inhibitory concentration (IC50, calcitriol only) for the drug, stimulating G1/G0 arrest, and causing a marked decrease in the mitochondrial transmembrane potential (30). In the current study, we have focused our attention on the antiangiogenic compound, cediranib, and its combination with calcitriol and low-calcemic vitamin D analog, calcipotriol, shown to be as potent as 1,25(OH)_2_D_3_ in human malignant melanoma cells (30).

## Materials and Methods

### Chemicals

1,25(OH)_2_D_3_ was purchased in Sigma-Aldrich (Merck KGaA, Darmstadt, Germany). Calcipotriol was a gift from the Pharmaceutical Research Institute (Warsaw, Poland). Cediranib (AZD2171) was purchased from Selleck Chemicals (Houston, TX, USA).

### Cell Culture

Human melanoma A375 cell line (CRL–1619), RPMI-7951 (HTB-66), MNT-1, and SK-MEL-28 were from the American Type Culture Collection (Manassas, VA, USA). The A375 cell line is derived from a skin melanoma of a 54-year-old female. It should be underlined that these cells carry two mutant genes, B-RAF and CDKN2, both associated with melanoma of sun-damaged skin (46). Since UV radiation is considered the most important environmental risk factor for cutaneous melanoma (47) and it is estimated that 60%–70% of cutaneous malignant melanomas are thought to be caused by ultraviolet (UV) radiation exposure (48), we therefore consider A375 melanoma cells as a particularly good model for our study. A375 cells were cultured in Dulbecco’s modified Eagle’s medium (DMEM, Sigma–Aldrich; Merck KGaA) supplemented with 10% fetal bovine serum (FBS) (Biological Industries, Israel) and 1% penicillin/streptomycin (Sigma–Aldrich; Merck KGaA) in an incubator with 5% CO_2_ at 37˚C. RPMI-7951 cells were cultured in Minimum Essential Medium Eagle, with Earle’s salts and non-essential amino acids (MEM, Sigma–Aldrich; Merck KGaA), supplemented with 10% FBS (Biological Industries, Israel), 1% penicillin/streptomycin (Sigma–Aldrich; Merck KGaA), 1 mM sodium pyruvate, and 2 mM L-glutamine (Sigma–Aldrich; Merck KGaA, both). MNT-1 cells were cultured in MEM (Sigma–Aldrich; Merck KGaA) Alpha Modification, supplemented with 20% FBS (Biological Industries, Israel), 1% penicillin/streptomycin, 10 mM HEPES, 2 mM L-glutamine, 1 mM sodium pyruvate, non-essential amino acids (Sigma–Aldrich; Merck KGaA, all listed before), and 10% AIM-V™ Medium (Thermofisher Scientific, Waltham, USA). SK-MEL-28 cells were cultured in MEM (Sigma–Aldrich; Merck KGaA) Alpha Modification, supplemented with 10% FBS (Biological Industries, Israel), 1% penicillin/streptomycin (Sigma–Aldrich; Merck KGaA), and 2 mM L-glutamine (Sigma–Aldrich; Merck KGaA). Appropriate medium supplemented with 5 times lower concentration of charcoal–stripped FBS was used for all procedures where the effects of vitamin D derivatives were examined (2% for A375, RPMI-7951, and SK-MEL-28 cell lines and 4% for MNT-1 cells).

### Proliferation Assay

The sulforhodamine B (SRB) assay was performed according to the procedure previously described (49). Briefly, the human melanoma A375 cells were seeded in 96–well plates (3,000 cells per well), cultured overnight, and then treated simultaneously with serial dilutions of cediranib (0.01–1,000 nM) and vitamin D analogs (calcitriol or calcipotriol) at 100 nM concentration, being tested for an additional 72 h. Cells were fixed with 10% trichloroacetic acid for 1 h at 4˚C. Following washing (5× with distilled water), the staining solution composed of 0.4% SRB (Sigma–Aldrich; Merck KGaA) in acetic acid was added to each well for 15 min, followed by washing with 1% acetic acid. The SRB dye was solubilized using a solution of 10 mM buffered Tris Base (pH 10.5) and the absorbance was measured at 570 nm using an Epoch™ microplate spectrophotometer (BioTek Instruments, Inc., Winooski, VT, USA).

### Cell Cycle Analysis

The cell cycle status was analyzed based on quantification of DNA content using flow cytometry. Melanoma cells were treated for 24 h with vitamin D compounds (calcitriol or calcipotriol) at 100 nM concentration, followed by 72 h incubation with cediranib at 500 or 1,000 nM concentration. Trypsinized human malignant melanoma cells together with cells from culture medium were fixed in 70% ethanol for 24–48 h at 4°C, then treated with ribonuclease to remove any contaminating RNA, and the DNA was stained with propidium iodide (PI; Sigma–Aldrich; Merck KGaA) for 30 min at 37°C. The fluorescence of the PI–stained cells was measured by flow cytometry (FACSCalibur™; Becton, Dickinson and Company, Franklin, Lakes, NJ, USA). The results were analyzed using the CellQuest™ Pro Software version 6.0 (Becton, Dickinson and Company) and expressed as a percentage of cells with DNA content corresponding to apoptotic/necrotic cells (subG1 fraction) or cells in G1, S, and G2/M phases of the cycle. **Supplementary Figure 1** presents row cytometrical data.

### Wound Closure Rate

A375 melanoma cells were seeded on an 8-well chamber slide (3 × 10^5^ cells per well) and were cultured overnight. Melanoma cells were pretreated with vitamin D compounds (calcitriol or calcipotriol) at 100 nM concentration. After 24 h, a mechanical wound was created by physical scraping using a pipette tip in a confluent cell monolayer. Cediranib at 500 or 1,000 nM concentration was diluted in a fresh medium and added to the cells for 72 h and cell migration process was observed. The experiment was carried out as a live imaging with Olympus cellVivo IX83 and cell free area was calculated as a percentage closure relative to original size [(wound area in μm^2^)*100/(original wound area in μm^2^)] with the Olympus cellSens software with use of TruAI technology.

### VEGFR2 Extracellular Expression

A375 melanoma cells were treated for 24 h with vitamin D compounds (calcitriol or calcipotriol) at 100 nM concentration, followed by 24 h incubation with cediranib at 500 or 1,000 nM concentration. Trypsinized human malignant melanoma cells at 1 × 10^6^ density were harvested by centrifugation and rinsed two times in 3 ml of incubation buffer (0.5% bovine serum albumin in PBS). Following 10 min blocking in the incubation buffer, cells were stained for 30 min at room temperature with primary antibody anti-VEGFR2 (Cell Signaling, cat. no. 2479, rabbit monoclonal, 1:200) dissolved in the incubation buffer. Following rinsing 2× in incubation buffer, cells were incubated for 30 min with the secondary antibody (goat anti-rabbit IgG ThermoFisher Scientific A11008, 1:500) diluted in the incubation buffer. Cells were rinsed 2× with incubation buffer, dissolved in 0.5 ml of PBS and analyzed cytometrically on FACSCalibur™ (Becton, Dickinson and Company, Franklin, Lakes, NJ, USA) using the CellQuest™ Pro Software version 6.0 (Becton, Dickinson and Company). The results were expressed as a fluorescence geometric mean.

### Immunoblotting

After preincubation either with 1,25(OH)_2_D_3_ or with calcipotriol at 100 nM concentration for 24 h, A375, SK-MEL-28, RPMI-7951 or MNT-1 melanoma cells were treated for an additional 24 h with cediranib at 500 or 1,000 nM concentration. Subsequently, cells were scraped and lysed in the presence of ice-cold RIPA buffer (Sigma–Aldrich; Merck KGaA) supplemented with protease inhibitor cocktail. Protein concentrations were determined by the Bradford assay. An equal amount of protein from each sample (40 μg) was loaded per lane, and proteins were resolved by SDS-PAGE (4%–20% Mini-PROTEAN^®^ TGX Stain-Free™ Protein Gels, Bio-Rad Laboratories, Hercules, CA, USA) and then transferred onto an Immun-Blot™ PVDF membrane (Bio-Rad Laboratories, Hercules, CA, USA). The membranes were incubated with primary antibodies: anti-VDR (mouse monoclonal, 1:1,000; Santa Cruz sc-13133), anti-VEGFR1 (rabbit polyclonal, 1:1,000; Cell Signaling Technology 2893), anti-VEGFR2 (rabbit monoclonal, 1:1,000; Cell Signaling Technology 2479), anti-PDGFR alpha (rabbit monoclonal, 1:1,000; Cell Signaling Technology 3174), anti-PDGFR beta (rabbit monoclonal, 1:1,000; Cell Signaling Technology 3169), or HRP-conjugated anti-β-actin antibody (mouse monoclonal, 1:10,000; Santa Cruz Biotechnology, sc-47778) overnight at 4°C. After three washes in TBST, secondary goat anti-rabbit antibodies conjugated to horseradish peroxidase (1:10,000; Santa Cruz Biotechnology, sc-2004) or secondary bovine anti-mouse antibodies conjugated to horseradish peroxidase (1:20,000; Santa Cruz Biotechnology, sc-2371) were added, and following incubation for 1 h at room temperature, blots were developed with Western Lightning^®^ Ultra chemiluminescent substrate (PerkinElmer, Inc. Waltham, MA, USA) according to the manufacturer’s protocol. Changes in protein level were assessed by densitometric scanning of the bands and corrected for β-actin loading control.

### Immunocytochemistry

A375 melanoma cells were seeded in 8-well chambers. Cells were preincubated for 24 h with vitamin D derivatives at 100 nM concentration and subsequently incubated for an additional 24 h with cediranib at 500 or 1,000 nM concentration. Following fixing with 4% paraformaldehyde (PFA) for 10 min at room temperature (RT), cells were permeabilized in 0.2% Triton X-100 solution in PBS for 10 min. Blocking was performed with 1% BSA in PBS for 30 min at RT. Following washing 3 × 5 min in PBS, primary antibodies were applied to the cells (VEGFR2 rabbit monoclonal, 1:200; Cell Signaling Technology 2479; EEA1 mouse monoclonal, 1:250, BD Biosciences 610457) and incubated at 4°C overnight. Following rinsing 3 × 5 min in PBS, slides were incubated with an appropriate secondary antibody (A11008 goat anti-rabbit IgG Alexa Fluor 488, 1:500; A11008 donkey anti-mouse IgG Alexa Fluor 594, 1:500, Life Technologies) for 1 h at RT. Following rinsing, cultures were counterstained with DAPI (Sigma–Aldrich; Merck KGaA). Images were collected with Olympus cellVivo IX83 and analyzed with Olympus cellSens software.

### RT-PCR

After preincubation either with 1,25(OH)_2_D_3_ or with calcipotriol at 100 nM concentration for 24 h, A375 melanoma cells were treated for an additional 24 h with cediranib at 500 or 1,000 nM concentration. Subsequently, total RNA was extracted by using the ExtractME^®^Total RNA Kit (Blirt, Poland, EM09.1-250), according to the manufacturer’s instructions. The concentration and purity of isolated RNA were measured by an EpochMicroplate Spectrophotometer (BioTek, USA). Extracted RNA was reverse transcribed and cDNA synthesized using RevertAid™ First Strand cDNA Synthesis Kit (Thermo Fisher Scientific Inc., USA). Real-Time PCR was performed using a StepOnePlus™ Real-Time PCR System (LifeTechnologies-Applied Biosystems, Grand Island, NY, USA) with RealTime AMPLIFYME SYBR™ Green No-ROX Mix (Blirt, Poland, AM01). All primers were purchased from Sigma-Aldrich (Merck KGaA). The expression of the genes was normalized by comparative -ΔΔCt method, using RPL37A as a housekeeping gene, followed by calibration (fold change) to normalized expression data of samples from control (ratio = 1). To ensure specificity of the PCR amplification, dynamic melting curve analysis was performed for all reactions. Primer sequences are summarized in **Table 1**.

**Table 1:** Primer sequences.

Gene	Forward primer 3’–5’	Reverse primer 5’–3’
RPL37A	TTCTGATGGCGGACTTTACC	CACTTGCTCTTTCTGTGGCA
VEGFR1	TCCAAGAAGTGACACCGAGA	TTGTGGGCTAGGAAACAAGG
VEGFR2	GACTTGGCCTCGGTCATTTA	ACACGACTCCATGTTGGTCA
PDGRFa	TGGATTGAACCCTGCTGATG	ATCAGCCTGCTT CATGTCCAT
PDGFRb	CACAATGACTCCCGTGGACTG	CATCATTAGGGAGGAAGCCCA
VEGFA	AAGGAGGAGGGCAGAATCAT	GCAGTAGCTGCGCTGATAGA
VEGFC	TGAACACCAGCACGAGCTAC	GCCTTGAGAGAGAGGCACTG
VEGFD	TGGAACAGAAGACCACTCTCATCT	GCAACGATCTTCGTCAAACATC
VDR	CCAGTTCGTGTGAATGATGG	GTCGTCCATGGTGAAGGA
CYP27B1	TGTTTGCATTTGCTCAGA	CCGGGAGAGCTCATACAG
CYP2R1	AGAGACCCAGAAGTGTTCCAT	GTCTTTCAGCACAGATGAGGTA
CYP3A4	AAGGCACCACCCACCTATGATACT	TACTTTGGGTCACGGTGAAGAGCA
CYP24A1	GCAGCCTAGTGCAGATTT	ATTCACCCAGAACTGTTG

### Statistical Analyses

Statistical analysis was performed using GraphPad Prism v 7.05 (GraphPad Software, San Diego, CA, USA) or Microsoft Excel. Data were subjected to Student’s *t*-test (for two groups), one-way or two-way analysis of variance and appropriate post-hoc test (the ANOVA Tukey’s or Sidak’s multiple comparison test). Data are expressed as mean of 3 to 5 independent experiments ± S.D (*n* = 2–6 in each). Differences are shown as significant at *p < 0.05,**p < 0.01, ***p < 0.001, or ****p < 0.0001 as indicated.

## Results

### Vitamin D Analogs Significantly Decrease Viability of A375 and SK-MEL-28 Melanoma Cells Treated With Cediranib

**Figure 1:**
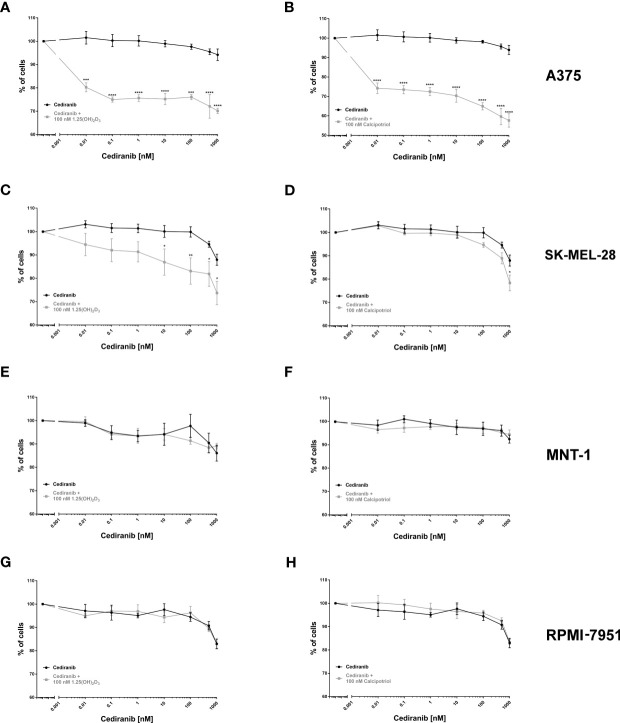
The effect of cediranib or its combination with vitamin D analogs [left column—1,25(OH)_2_D_3_; right column—calcipotriol] on the proliferation of human malignant melanoma A375, MNT-1, RPMI-7951, and SK-MEL-28 cells [**(A, B)**—A375; **(C, D)**—SK-MEL-28; **(E, F)**—MNT-1 and **(G, H)**—RPMI-7951 cell lines]. The cells were treated with serial dilutions (0.01–1,000 nM) of cediranib alone or in combination either with 1,25(OH)_2_D_3_ or with calcipotriol for 72 h. The same cediranib data are plotted in each graph from the same melanoma cell line, except for MNT-1 cells. Data are shown as mean from three or four independent experiments (*n* = 4–6 in each) ± SEM. Statistical significance between plots (between relevant concentrations of cediranib alone or with vitamin D) was estimated using two–way ANOVA and presented as *p < 0.05; **p < 0.01, ***p < 0.001, or ****p < 0.0001.

As established by SRB proliferation assay, cediranib alone inhibited A375 melanoma cell proliferation maximally about 6% at 1,000 nM concentration during 72 h of incubation (**Figures 1A, B**). However, simultaneous treatment with cediranib and 1,25(OH)_2_D_3_ or calcipotriol, at 100 nM concentration, resulted in a profound decrease in the proliferation of melanoma cells. The effect of vitamin D derivatives varied as to the level of maximal inhibition of melanoma cell proliferation, which ranged from approximately 30% for cediranib and 1,25(OH)_2_D_3_ (**Figure 1A**) to 43% for cediranib and calcipotriol (**Figure 1B**), *p* < 0.0001 both. Similar effects were observed in SK-MEL-28 melanoma cells. Cediranib alone inhibited proliferation of the cells maximally about 12% at 1,000 nM under experimental conditions used (**Figures 1C, D**). Simultaneous treatment with cediranib and 1,25(OH)_2_D_3_ or calcipotriol, at 100 nM concentration, resulted in further decrease in the proliferation of melanoma cells. The effect of vitamin D derivatives varied as to the level of maximal inhibition of melanoma cell proliferation, which ranged from approximately 26% for cediranib and 1,25(OH)_2_D_3_ (**Figure 1C**) to 22% for cediranib and calcipotriol (**Figure 1D**), *p* < .05 both. On the other hand, treatment of MNT-1 and RPMI-7951 melanoma cells with cediranib in the presence of 1,25(OH)_2_D_3_ (**Figures 1E–H**) or calcipotriol did not show additive effect of co-treatment. Cediranib alone inhibited proliferation of these melanoma cells maximally about 11% or 18%, respectively, at 1,000 nM concentration during 72 h of incubation.

### Vitamin D Derivatives Trigger G2/M Cell Cycle Arrest in A375 Malignant Melanoma Cells Treated With Cediranib

**Figure 2:**
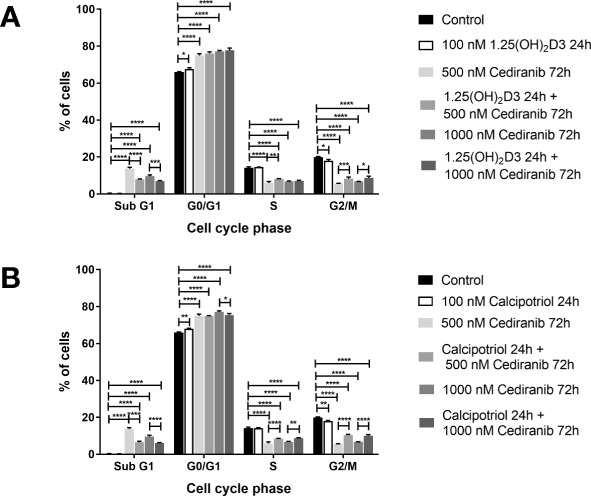
The effect of 24-h preincubation with 1,25(OH)_2_D_3_ **(A)** or calcipotriol **(B)** at 100 nM concentration on the distribution of human malignant melanoma A375 cells treated for 72 h with cediranib throughout the phases of the cell cycle (SubG1—apoptotic/necrotic cells, G1—growth, S—DNA synthesis, G2/M—preparation for mitosis/mitosis). Cells were harvested, stained with propidium iodide, and analyzed by flow cytometry. The data are presented as the mean ± standard deviation (*n* = 3). The same control and cediranib data are plotted in each graph. Statistical significance was estimated using two–way ANOVA followed by Tukey’s multiple comparison test and presented as *p < 0.05; **p < 0.01; ***p < 0.001, ****p < 0.0001. The results are representative of four experiments.

Since the most profound effect of vitamin D analogs to the inhibition of melanoma cell proliferation treated with cediranib was observed in A375 cells, this line was used as a model for further detailed analysis. In agreement with our previous studies (15, 44), treatment of A375 melanoma cells with vitamin D resulted in G0/G1 (G0/G1—stationary/growth phase) cell cycle arrest (**Figures 2A, B**). G0/G1 arrest was observed also in melanoma cells treated with cediranib, *p* < 0.0001 (**Figure 2**). Additionally, we noticed an increase in the number of SubG1 cells, indicating induction of apoptosis by cediranib in melanoma cells, *p* < 0.0001 (**Figure 2**). To investigate the mechanism of proliferation inhibition of melanoma A375 cells by the combination of vitamin D analogs with cediranib, melanoma cells were pretreated either with 1,25(OH)_2_D_3_ (**Figure 2A**) or with calcipotriol (**Figure 2B**) at 100 nM concentration for 24 h and then incubated with cediranib at 500 or 1,000 nM for an additional 72 h. Preincubation of melanoma cells with 1,25(OH)_2_D_3_ (**Figure 2A**) prior to cediranib treatment for 72 h resulted in an increase in the percentage of cells in the G2/M phase (preparation for mitosis/mitosis) in comparison to cells without pretreatment, *p* < 0.001 for cediranib at 500 nM concentration and *p* < 0.05 for cediranib at 1,000 nM concentration (**Figure 2A**), which was accompanied by a proportional decrease in the number of SubG1 cells (SubG1—apoptotic/necrotic cells). Similar results were observed for calcipotriol (**Figure 2B**); however, we noticed an increase in the percentage of cells not only in the G2/M phase, but also in the S phase, in comparison to cells without pretreatment.

### Pretreatment With Vitamin D Derivatives Significantly Decreases Mobility of A375 Melanoma Cells Treated With Cediranib

**Figure 3:**
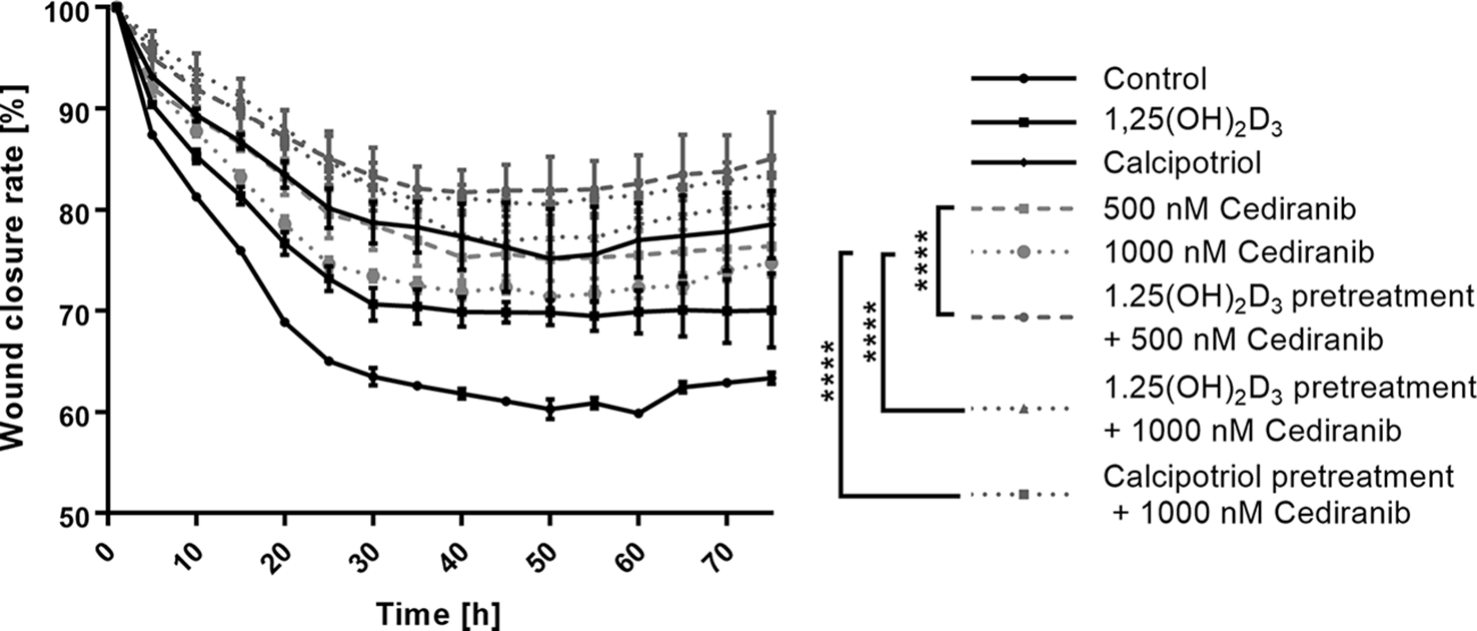
The effect of 24-h preincubation with vitamin D analogs at 100 nM concentration on the rate of a wound closure in A375 human malignant melanoma cells treated for 72 h with cediranib either at 500 or at 1,000 nM concentration. The cell-free area of each wound was measured at the different time points, every 30 min for 72 h as a live imaging in Olympus cell Vivo IX 83, and results were calculated in % as a wound closure rate with the Olympus cell Vivo IX 83 software. Statistical values were calculated with one-way analysis of variance and Tukey’s post-hoc test and presented as ****p < 0.0001.

Cellular motility and migration are well-established hallmarks of malignant tumors spreading their metastases (50). We recorded therefore migration of A375 melanoma cells and wound closure live with Olympus cellVivo IX83 every 30 min for 72 h and cell free area was calculated as a percentage of closure relative to its original size. The wound closure curves (**Figure 3**) revealed that non-treated malignant melanoma A375 cells approached up to 38% closure and migrate faster than cells from any treatment groups (*p* < 0.0001 for any treatment group vs. control, not marked in **Figure 3**). In agreement with our previous study (15), we observed that 1,25(OH)_2_D_3_ efficiently inhibited melanoma cell migration (*p* < 0.0001 vs. control, not marked in **Figure 3**) during 72 h, leaving approximately 70% of the wound original size. Interestingly, calcipotriol was even more efficient than 1,25(OH)_2_D_3_, leaving as much as 81.5% of the wound original size. Curiously, cediranib at both tested concentrations, 500 and 1,000 nM, inhibited melanoma cell migration to a similar extent, leaving approximately 74%–75% of the wound original size. It should be emphasized, however, that vitamin D pretreatment profoundly diminished cellular mobility in melanoma cells treated with cediranib. The most efficient reduction of melanoma cells mobility was observed in cells 24 h pretreated with 1,25(OH)_2_D_3_ and incubated subsequently for 72 h with cebiranib at 500 nM, in which the wound area was reduced by only 15% [*p* < 0.0001 for melanoma cells 1,25(OH)_2_D_3_ pretreated and incubated with cediranib at 500 nM concentration vs. 500 nM cediranib alone; **Figure 3**], giving a further significant 10% reduction in cellular mobility as compared to monotreatment with cediranib. Substantial 8% reduction in cellular mobility was observed also in melanoma cells pretreated with calcipotriol compared to monotreatment with cediranib.

### Vitamin D Derivatives Increase the Extracellular Expression of VEGFR2 in A375 Malignant Melanoma Cells Treated With Cediranib

**Figure 4:**
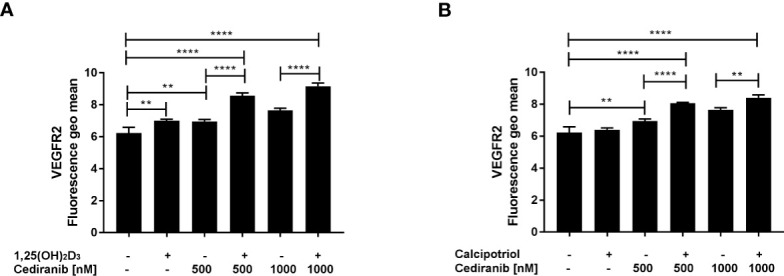
The effect of 24-h preincubation with 1,25(OH)2D3 **(A)** or calcipotriol **(B)** at 100 nM concentration on the extracellular expression of the VEGFR2 in A375 melanoma cells treated subsequently for 24 h with cediranib at 500 or 1,000 nM concentration. Cells were stained with appropriate antibody (see *Materials and Methods* section) and analyzed cytometrically. The data are presented as the mean ± standard deviation (*n* = 3). The same control and cediranib data are plotted in each graph. Statistical significance was estimated using one–way ANOVA followed by Tukey’s multiple comparison test and presented as **p < 0.01 or ****p < 0.0001. The results are representative of three experiments.

Since cediranib is a small-molecule inhibitor of several tyrosine kinases, including VEGFR1 and VEGFR2, of which the latter seems to play a predominant role (51), we investigated therefore whether vitamin D preincubation will affect the extracellular expression of VEGFR2 in A375 melanoma cells exposed to cediranib. We noticed that cediranib alone did not influence the extracellular expression of VEGFR2 in A375 melanoma cells during 24-h incubation (**Figure 4**). However, the extracellular expression of VEGFR2 increased significantly in melanoma cells pretreated either with 1,25(OH)_2_D_3_ (**Figure 4A**) or with calcipotriol (**Figure 4B**) for 24 h as compared to monotreatment with cediranib or to control cells.

### Vitamin D Derivatives Upregulate VEGFR2 Protein Level in Malignant Melanoma Cells Treated With Cediranib

**Figure 5:**
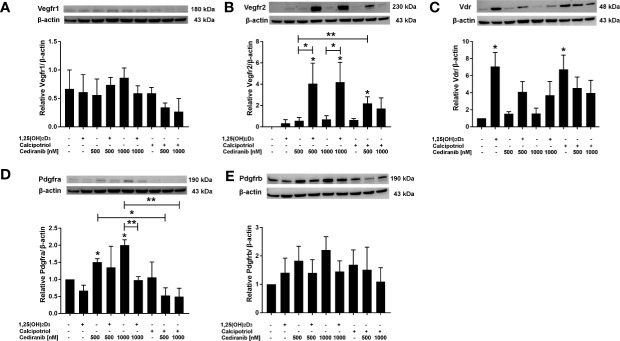
The effect of 24-h preincubation with vitamin D analogs at 100 nM concentration on VEGFR1 **(A)**, VEGFR2 **(B)**, VDR **(C)**, PDGFRa **(D)**, and PDGFRb **(E)** protein level in A375 melanoma cells treated subsequently for 24 h with cediranib at 500 or 1,000 nM concentration. Protein levels were measured by Western blotting, with β-actin used as a control. Data are shown as mean from three independent experiments ± SEM. *p < 0.05 and **p < 0.01 vs. untreated control or between the two groups indicated by the bracket.

Next, we checked whether the preincubation of A375 melanoma cells with vitamin D derivatives affected the protein level of VEGFR1, VEGFR2, PDGFRa, PDGFRb, or VDR after subsequent treatment with cediranib (**Figure 5**). No significant effect was observed as to the VEGFR1 or PDGFRb protein level neither by 1,25(OH)_2_D_3_, nor by cediranib under the experimental conditions used (**Figures 5A, E**, respectively). We also noticed that the expression of VEGFR2 at the protein level was not changed by cediranib alone (**Figure 5B**). However, we observed a significant increase in VEGFR2 protein level in melanoma cells pretreated with vitamin D (although in case of calcipotriol, only with cediranib at 500 nM concentration). Both vitamin D derivatives increased the VDR protein level (**Figure 5C**). Cediranib alone increased the protein level of PDGFRa (*p* < 0.05), while preincubation with 1,25(OH)_2_D_3_ reversed that effect for cediranib at 1,000 nM concentration, *p* < 0.01 (**Figure 5D**).

**Figure 6:**
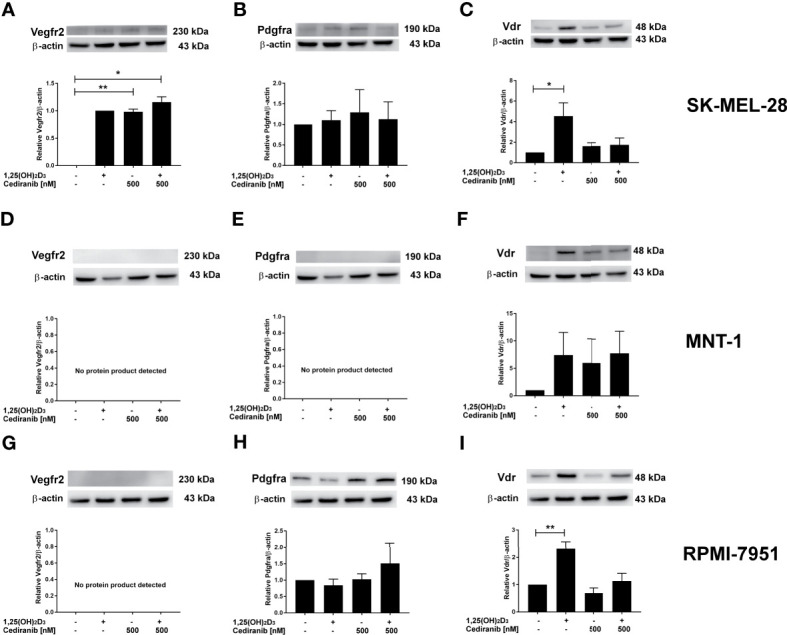
The effect of 24-h preincubation with 1,25(OH)_2_D_3_ at 100 nM concentration on VDR **(C, F, I)**, PDGFRa **(B, E, H)**, and VEGFR2 **(A, D, G)** protein level in SK-MEL-28 **(A–C)**, MNT-1 **(D–F)**, and RPMI-7951 **(G–I)** melanoma cells treated subsequently for 24 h with cediranib at 500 nM concentration. Protein levels were measured by Western blotting, with β-actin used as a control. Data are shown as mean from three independent experiments ± SEM. Statistical significance was estimated using one-way ANOVA followed by Tukey’s multiple comparison test and presented as *p < 0.05 and **p < 0.01 vs. untreated control or between the two groups indicated by the bracket.

Since we observed the upregulation of VEGFR2, at both protein level and its extracellular expression, under experimental conditions, concurrently with a very desirable decrease in A375 melanoma cell proliferation and mobility, we hypothesized whether the presence of VEGFR2 protein or its level could potentially influence the extent to which 1,25(OH)_2_D_3_ may enhance the cytotoxic effect of cediranib in MNT-1, RPMI-7951, and SK-MEL-28 melanoma cells. We checked, therefore, whether the preincubation of aforementioned melanoma cell lines with 1,25(OH)_2_D_3_ affected the protein level of VEGFR2, PDGFRa, or VDR, after subsequent treatment with cediranib (**Figure 6**). No significant effect was observed as to the VDR protein level neither by 1,25(OH)_2_D_3_, nor by cediranib under the experimental conditions used in MNT-1 melanoma cells (**Figure 6**). However, we observed a significant increase in VDR protein level in RPMI-7951 and SK-MEL-28 melanoma cells treated with 1,25(OH)_2_D_3_ (**Figures 6C, I**). No significant effect was observed as to the PDGFRa protein level neither by 1,25(OH)_2_D_3_, nor by cediranib under the experimental conditions used in RPMI-7951 and SK-MEL-28 melanoma cells (**Figures 6B, H**); what is more, we did not detect any PDGFRa protein product in MNT-1 melanoma cells (**Figure 6E**). Interestingly, we did not detect any VEGFR2 protein product neither in MNT-1, nor in RPMI-7951 melanoma cells (**Figures 6D, G**). However, we observed a significant increase in VEGFR2 protein level in SK-MEL-28 melanoma cells treated with cediranib alone or cediranib with 1,25(OH)_2_D_3_ (**Figure 6A**), which underlines the key role of VEGFR2 in an interaction between vitamin D and cediranib.

**Figure 7:**
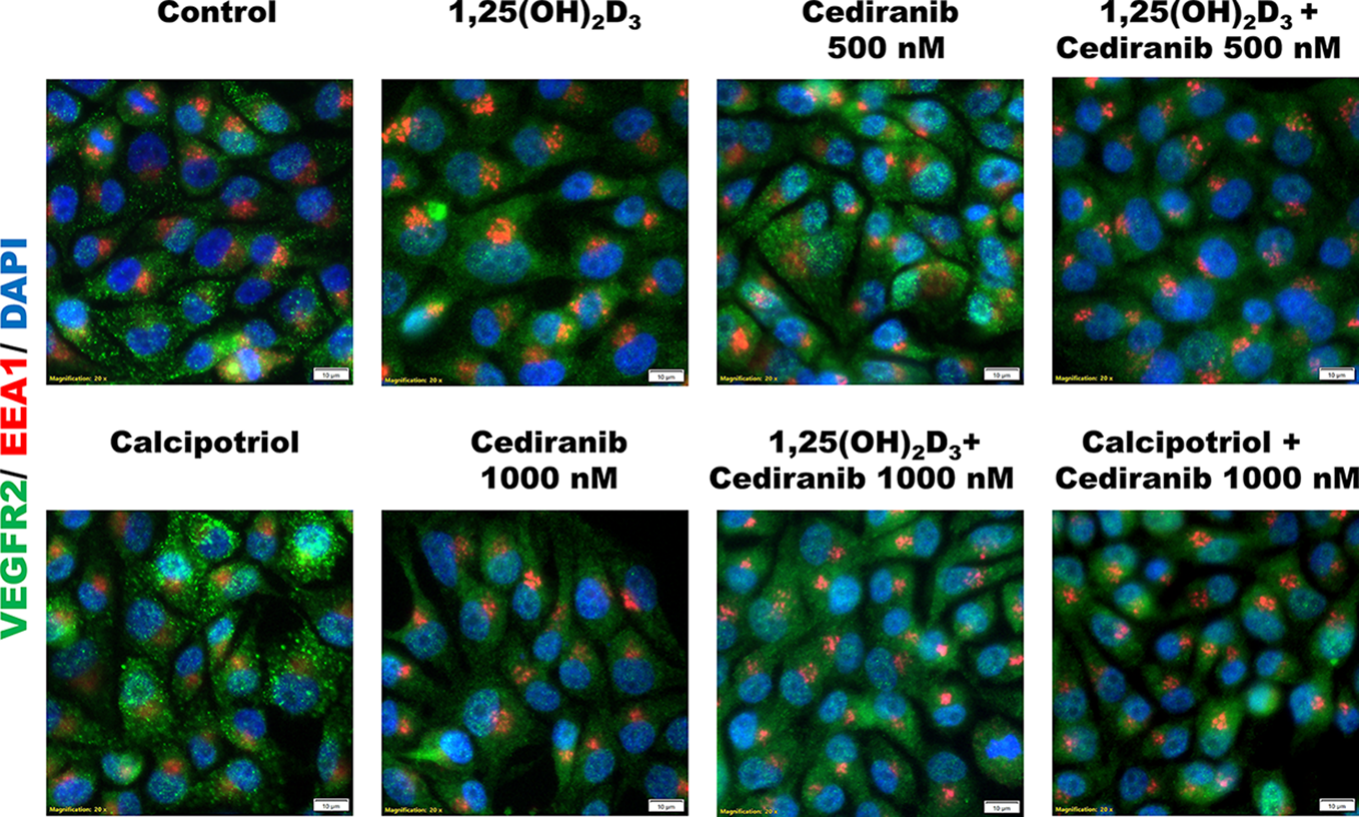
Immunofluorescent detection of VEGFR2 (green) or EEA1 (red) in A375 melanoma cells. Melanoma cells were preincubated with vitamin D derivatives for 24 h and subsequently treated with cediranib at 500 or 1,000 nM concentration for another 24 h. Nuclei were counterstained with DAPI (shown in blue). Magnification 200×.

To further explore the mechanism underlying the observed increase of VEGFR2 protein level by vitamin D in A375 melanoma cells treated with cediranib, we checked whether this protein is sequestered in early endosomes for potential recycling or degradation, as suggested recently (52). We did not observe, however, any co-localization of VEGFR2 and EEA1, which is a marker of early endosomes (**Figure 7**).

### Vitamin D Derivatives Modulate Expression of VEGFR2, VEGFA, PDGFRa, and PDGFRb at the mRNA Level in A375 Malignant Melanoma Cells Treated With Cediranib

**Figure 8:**
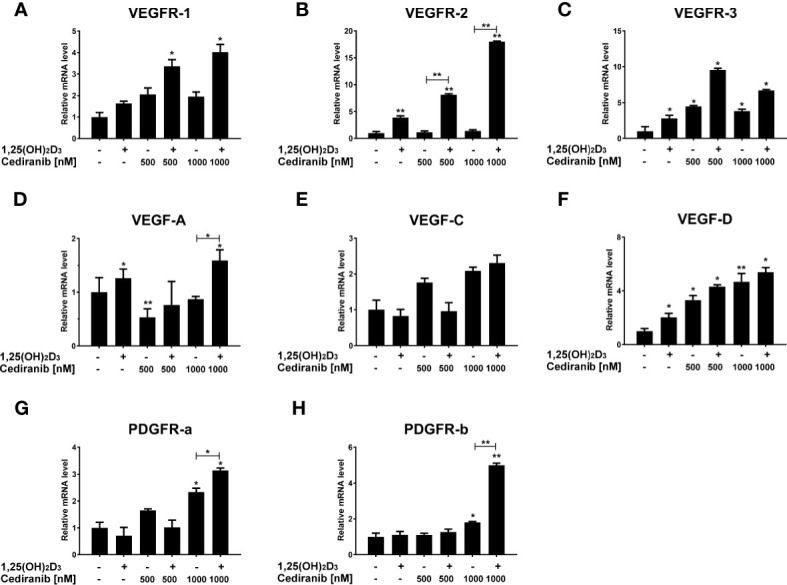
The effect of 24-h preincubation with 1,25(OH)_2_D_3_ at 100 nM concentration on VEGFR1 **(A)**, VEGFR2 **(B)**, VEGFR3 **(C)**, VEGFA **(D)**, VEGFC **(E)**, VEGFD **(F)**, PDGFRa **(G)**, and PDGFRb **(H)** gene expression in A375 melanoma cells treated subsequently for 24 h with cediranib at 500 or 1,000 nM concentration. mRNA levels were measured by qPCR. The results are representative of three experiments carried out in duplicate. *p < 0.05 and **p < 0.01 vs. untreated control or between the two groups indicated by the bracket.

In order to verify the aforementioned changes in protein level, the impact of 1,25(OH)_2_D_3_ on the expression of selected VEGF-associated genes was tested in melanoma cells treated with cediranib (**Figure 8**). Although cediranib alone did not influence mRNA level for VEGFR1 (**Figure 8A**), we observed an increase in mRNA level in melanoma cells treated with the combination of 1,25(OH)_2_D_3_ and cediranib at both tested concentrations (*p* < 0.05 vs. control). No significant effect of cediranib alone was observed on the mRNA level of VEGFR2; however, consistent with immunoblotting described above, we observed a marked increase in VEGFR2 mRNA level in melanoma cells pretreated with 1,25(OH)_2_D_3_ (*p* < 0.01, **Figure 8B**). Both 1,25(OH)_2_D_3_ and cediranib resulted in an increase in mRNA level for VEGFR3 (*p* < 0.05, **Figure 8C**), with an increasing trend in melanoma cells pretreated with vitamin D subsequently exposed to cediranib, yet without statistical significance in the latter. Interestingly, cediranib alone at 500 nM concentration decreased the mRNA level for VEGF-A (*p* < 0.01, **Figure 8D**), while pretreatment of A375 melanoma cells with 1,25(OH)_2_D_3_ resulted in an increase in the relevant mRNA in melanoma cells incubated subsequently with cediranib at 1,000 nM concentration (*p* < 0.05). No significant effect was observed in the expression of VEGF-C under the experimental conditions used (**Figure 8E**). mRNA level for VEGF-D was elevated by both 1,25(OH)_2_D_3_ and cediranib (*p* < 0.05, **Figure 8F**), and it was elevated also in melanoma cells pretreated with vitamin D. Finally, we observed an increase in mRNA level for PDGFRa and PDGFRb in melanoma cells treated with cediranib at 1,000 nM concentration (*p* < 0.05, **Figures 8G, H**, respectively); the effect was further exacerbated by vitamin D pretreatment (*p* < 0.05 for PDGFRa and *p* < 0.01 for PDGFRb vs. monotreatment).

**Figure 9:**
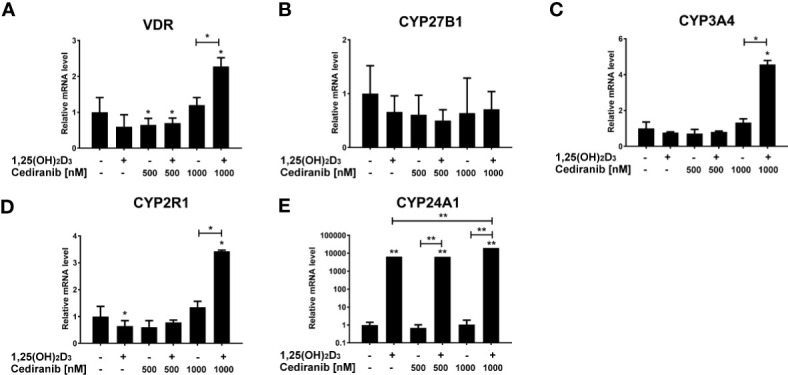
The effect of 24-h preincubation with 1,25(OH)_2_D_3_ at 100 nM concentration on VDR **(A)**, CYP27B1 **(B)**, CYP3A4 **(C)**, CYP2R1 **(D)**, and CYP24A1 **(E)** gene expression in A375 melanoma cells treated subsequently for 24 h with cediranib at 500 or 1,000 nM concentration. mRNA levels were measured by qPCR. The results are representative of three experiments carried out in duplicate. *p < 0.05 and **p < 0.01 vs. untreated control or between the two groups indicated by the bracket.

Then, we also analyzed the expression of several vitamin D-related genes in the experimental conditions used. We observed that VDR mRNA level was elevated in A375 melanoma cells pretreated with vitamin D and subsequently incubated with cediranib at 1,000 nM concentration (*p* < 0.05, **Figure 9A**). No significant effect was observed in the expression of CYP27B1 under the experimental conditions used (**Figure 9B**). Cediranib treatment resulted, however, in an increase in mRNA level for CYP3A4 and CYP2R1 in melanoma cells pretreated with 1,25(OH)_2_D_3_ (*p* < 0.05 vs. control and vs. monotreatment, **Figures 9C, D**, respectively). Lastly, consistent with our previous results (30) and literature data (53), we observed a marked increase in mRNA level for CYP24A1 (*p* < 0.01, **Figure 9E**) in melanoma cells treated with 1,25(OH)_2_D_3_. Interestingly, the effect was invariably observed in cells treated subsequently with cediranib. In fact, the mRNA level of CYP24A1 was the highest in melanoma cells treated with cediranib following vitamin D pretreatment (*p* < 0.01 vs. monotreatment).

## Discussion

Advanced metastatic melanoma is widely known as one of the most aggressive skin malignancies. Regardless of improvements in the recent decade, a remarkable proportion of patients still fail to respond to the therapy or will relapse over time (4). Increased effort in search for the neoadjuvant and adjuvant settings may therefore help to improve long-term outcomes for melanoma-suffering patients. In that field, vitamin D seems to be a promising and reasonable remedy, acting as both a chemopreventive and therapeutic agent (47). Firstly, it is well documented that vitamin D protects against DNA damage (54, 55) and therefore against UV-induced carcinogenesis (56–58), since UV is considered as the major environmental risk factor for melanoma development (47). Secondly, vitamin D deficiency is a well-established cancer risk factor (59), while vitamin D supplementation was shown to reduce the incidence of advanced and fatal cancer (18). What is more, vitamin D deficiency is associated with higher Breslow thickness and mortality in melanoma patients (60). Furthermore, an increase in 25-hydroxyvitamin D_3_ level in vitamin D-deficient melanoma patients already undergoing treatment improved their outcomes in comparison to individuals who remained vitamin D deficient (61). Finally, an inverse correlation between vitamin D receptor, VDR, and 1α-hydroxylase (CYP27B1), the enzyme responsible for the synthesis of the biologically active form of vitamin D, was documented with melanoma progression and disease outcome (25, 26).

Our previous study revealed that two vitamin D analogs, calcitriol and low calcemic calcipotriol, exhibited modulatory effects on the A375 melanoma cells treated with dacarbazine, decreasing the half maximal inhibitory concentration (IC50, calcitriol only) for the drug, stimulating G1/G0 arrest, and causing a marked decrease in the mitochondrial transmembrane potential under given experimental conditions (30). Since the process of angiogenesis is crucial for growth, progression, and metastasis of the majority of solid tumors, including melanomas (62, 63), in the current study, the effects of cediranib, an oral tyrosine kinase inhibitor (TKI) of VEGFR1-3, PDGFR, and c-KIT (64), used in combination either with 1,25(OH)_2_D_3_ or with low-calcemic analog calcipotriol, were tested in the same A375 human malignant melanoma cell line, carrying the BRAF^V600E^ mutation, very common in melanoma patients (65), which was shown to be pro-angiogenic in several human tumors (66). Selected experiments were also carried out in MNT-1, RPMI-7951, and SK-MEL-28 melanoma cell lines. Cediranib as a single agent is associated predominantly with hypertension, diarrhea, dysphonia, and proteinuria, as shown in a phase I study (67). The most frequent non-hematologic adverse events observed in patients with metastatic or recurrent malignant melanoma treated with cediranib in a phase II study were hypertension (78%), fatigue (69%), diarrhea (69%), and anorexia and nausea (each 57%) (68). It should be emphasized that melanomas express high levels of VEGF, VEGFR1, VEGFR2, and VEGFR3, which is further associated with poor prognosis (69, 70). Quite unexpectedly, it was shown that adjuvant treatment with bevacizumab, an anti-VEGF monoclonal antibody, after resection of high-risk melanoma significantly improves disease-free interval, rather than overall survival (66). In fact, bevacizumab as a monotherapy does not offer any significant survival benefit over traditional therapies (71, 72). Therefore, antiangiogenic therapies in melanoma are rather supportive to other forms of treatment. However, various configurations of combination therapies with antiangiogenic bevacizumab against melanoma are currently being investigated in clinical trials (73). Very interestingly, as documented recently by Atzori (74), VEGFR1 inhibition might potentiate the effects of vemurafenib-based therapies for melanoma treatment and, what is more, counteract resistance development to this BRAF inhibitor, since the latter was associated with higher expression of VEGF receptors. Although cediranib alone was not sufficiently effective as a first-line therapy in untreated patients with metastatic or recurrent malignant melanoma, as shown in a phase II study (68), of the 17 patients evaluable for response, 2 patients had stable disease >/= 6 months, and the disease was stable in 8 patients and progressive in 9 patients, with no objective responses seen. Still, the authors concluded that the potential of cediranib may be enhanced in combination with other agents (68). Furthermore, it was shown recently on patient-derived organoid models of endometrial cancer that cediranib but not bevacizumab synergizes with chemotherapy, decreasing cell viability when combined with paclitaxel as compared to treatment with paclitaxel alone (75). Currently, there is an ongoing phase I clinical trial, NCT01364051, for patients with clinically unresectable solid tumors, including stage IV cutaneous melanoma and malignant melanoma, and for whom there is no standard therapy, in which patients are receiving cediranib with selumetinib, an oral MEK 1/2 inhibitor. In our experiments, vitamin D derivatives were used at 100 nM concentration, corresponding to the optimal serum 25(OH)D_3_ level (75–125 nM) (76), which is used in clinic as a biomarker of vitamin D status. We have shown that supplementation with vitamin D improves the effectiveness of anti-angiogenic compound, cediranib, against A375 and SK-MEL-28 human melanoma cells, as we observed a marked decrease in melanoma cell proliferation (in both lines), G2/M cell cycle arrest, and a significant decrease in melanoma cell mobility (tested only in A375 melanoma cells). A similar observation was recently described in Hec50 cells, an endometrial adenocarcinoma, in which the combination of paclitaxel and cediranib produced a profound increase in the accumulation of cells in mitosis as assessed by the percentage of cells in G2/M by flow cytometry compared to paclitaxel alone (75). On the other hand, we did not observe any influence of vitamin D on proliferation of MNT-1 and RPMI-7951 melanoma cells treated with cediranib. The study of Atzori (74) suggested that VEGFR1 upregulation might contribute to melanoma progression and spreading. Overexpressed VEGFR2 in gastric cancer cells increased cellular proliferation and invasion *in vitro* as well as tumor formation in xenograft models (77). The pro-metastatic role of VEGFR2 was also postulated in osteosarcoma (78). Surprisingly, we observed the upregulation of VEGFR2 in experimental conditions used concurrently with a very desirable decrease in melanoma cell proliferation and mobility. Interestingly, it seems that vitamin D derivatives enhance cediranib efficacy by modulation of VEGFR2 expression in melanoma cells, as we observed a significant increase in VEGFR2 level at both protein and mRNA levels, along with the extracellular VEGFR2 expression, in vitamin D-pretreated A375 melanoma cells incubated further with cediranib. Thus, the extent to which vitamin D exacerbates cytotoxicity of cediranib against melanoma cells seems to depend firstly on the presence of VEGFR2 in these cells and secondly on its level. The most profound increase in cediranib cytotoxicity by supplementation with vitamin D was observed in A375 melanoma cells, in which we noticed the upregulation of VEGFR2, at both the protein and mRNA level, as well as its extracellular expression, in experimental conditions used, concurrently with a very desirable decrease in melanoma cell proliferation and mobility. The VEGFR2 protein level was several times higher in vitamin D-pretreated cells compared to monotreatment with cediranib. Similarly, in SK-MEL-28 cells, simultaneous treatment with cediranib and 1,25(OH)_2_D_3_ or calcipotriol, at 100 nM concentration, resulted in a further decrease in the proliferation of these melanoma cells, which was accompanied by an increase in the protein level of VEGFR2 compared to control cells in experimental conditions used, although there was no difference in the VEGFR2 protein level compared to monotreatment with cediranib. On the other hand, in MNT-1 and RPMI-7951 melanoma cells, in which we did not observe any enhancement of cediranib cytotoxicity by supplementation with vitamin D, we did not detect any VEGFR2 protein in the experimental conditions used. It should be noted that VEGFR2 is considered a predominant receptor triggering VEGF signaling in cells (73, 79). Out of the three primary VEGF receptors, VEGFR2 is considered the dominant effector and the most relevant in the metastatic melanoma microenvironment, although the study of Molhoek and coworkers showed that a relatively low percentage of melanoma cells express VEGFR2 (80). However, yet another study underlines that it is VEGFR2 that might be a new prognostic marker in malignant melanoma (81).

Possibly, the astonishing upregulation of VEGFR2 observed in A375 and SK-MEL-28 melanoma cells in our experimental conditions may be considered as an adaptive strategy activated by vitamin D, in which increased availability of VEGFR2 on the melanoma cell surface potentiates the response to its inhibitor, cediranib, or elevated expression of the receptor is a response to inhibition of the downstream signaling from the receptor.

We also observed an increase in VDR mRNA level as well as CYP3A4 and CYP2R1 in A375 cells, the enzymes responsible for 25-hydroxylation of vitamin D en route to its final activation, in melanoma cells treated with cediranib at 1,000 nM concentration, which were 1,25(OH)_2_D_3_ pretreated, underlining an intensified vitamin D activation in these conditions (*p* < 0.05, **Figure 9**). Thus, it is possible that cediranib actually improves the sensitivity of cells to vitamin D.

In conclusion, although recent innovative immunotherapies and targeted therapies have vastly ameliorated the management of metastatic melanoma, in light of impending resistance development, more effective strategies for treatment of melanoma patients are still urgently needed. We demonstrated that vitamin D derivatives hold promise as novel adjuvant candidates to conquer melanoma, which may be considered for clinical applications, especially in vitamin D-deficient melanoma patients, as they are widely available, non-toxic, and relatively inexpensive. However, further extensive and complex studies are needed to assess their potential expected benefits for melanoma patients.

## Data Availability Statement

The datasets used and/or analyzed during the current study are available from the corresponding author on reasonable request.

## Author Contributions

AP and MAŻ conceived, designed, and supervised the study. AP, FBP, JIN, and JMW performed the experiments. AP and MAŻ analyzed the data. AP and MAŻ wrote the manuscript. All authors contributed to the article and approved the submitted version.

## Funding

This study was supported by grant no. MN 01–0250/08/280 from the Medical University of Gdansk (Gdansk, Poland) to AP. The authors would like also to thank São Paulo Research Foundation (FAPESP) for the grant support: 2017/17600-1.

## Conflict of Interest

The authors declare that the research was conducted in the absence of any commercial or financial relationships that could be construed as a potential conflict of interest.

## Publisher’s Note

All claims expressed in this article are solely those of the authors and do not necessarily represent those of their affiliated organizations, or those of the publisher, the editors and the reviewers. Any product that may be evaluated in this article, or claim that may be made by its manufacturer, is not guaranteed or endorsed by the publisher.

## Supplementary Material

Supplementary Figure 1 The effect of 24 hours preincubation with vitamin D analogs at 100 nM concentration on the distribution of human malignant melanoma A375 cells treated for 72 hours with cediranib throughout phases of the cell cycle (M1: SubG1—apoptotic/necrotic cells, M2: G1—growth, M3: S—DNA synthesis, M4: G2/M—preparation for mitosis/mitosis). Cells were harvested, stained with propidium iodide and analyzed by Flow Cytometry. Presented panels show representative data plots for relevant treatments.

Supplementary Figure 2 The effect of 24 hours preincubation with vitamin D analogs at 100 nM concentration on the extracellular expression of the VEGFR2 in A375 melanoma cells treated subsequently for 24 hours with cediranib at 500 or 1000 nM concentration. Cells were stained with appropriate antibody (see Materials and methods section) and analyzed cytometrically. Presented panels show representative data plots for relevant treatments.
