# Supplementary material for: Vitamin D Enhances Anticancer Properties of Cediranib, a VEGFR Inhibitor, by Modulation of VEGFR2 Expression in Melanoma Cells
Source: Front Oncol. 2021 Dec 24;11:763895. doi: 10.3389/fonc.2021.763895 (PMC8740239; doi:10.3389/fonc.2021.763895)
Supplement: Supplementary Figure 1 [file Image_1.pdf]

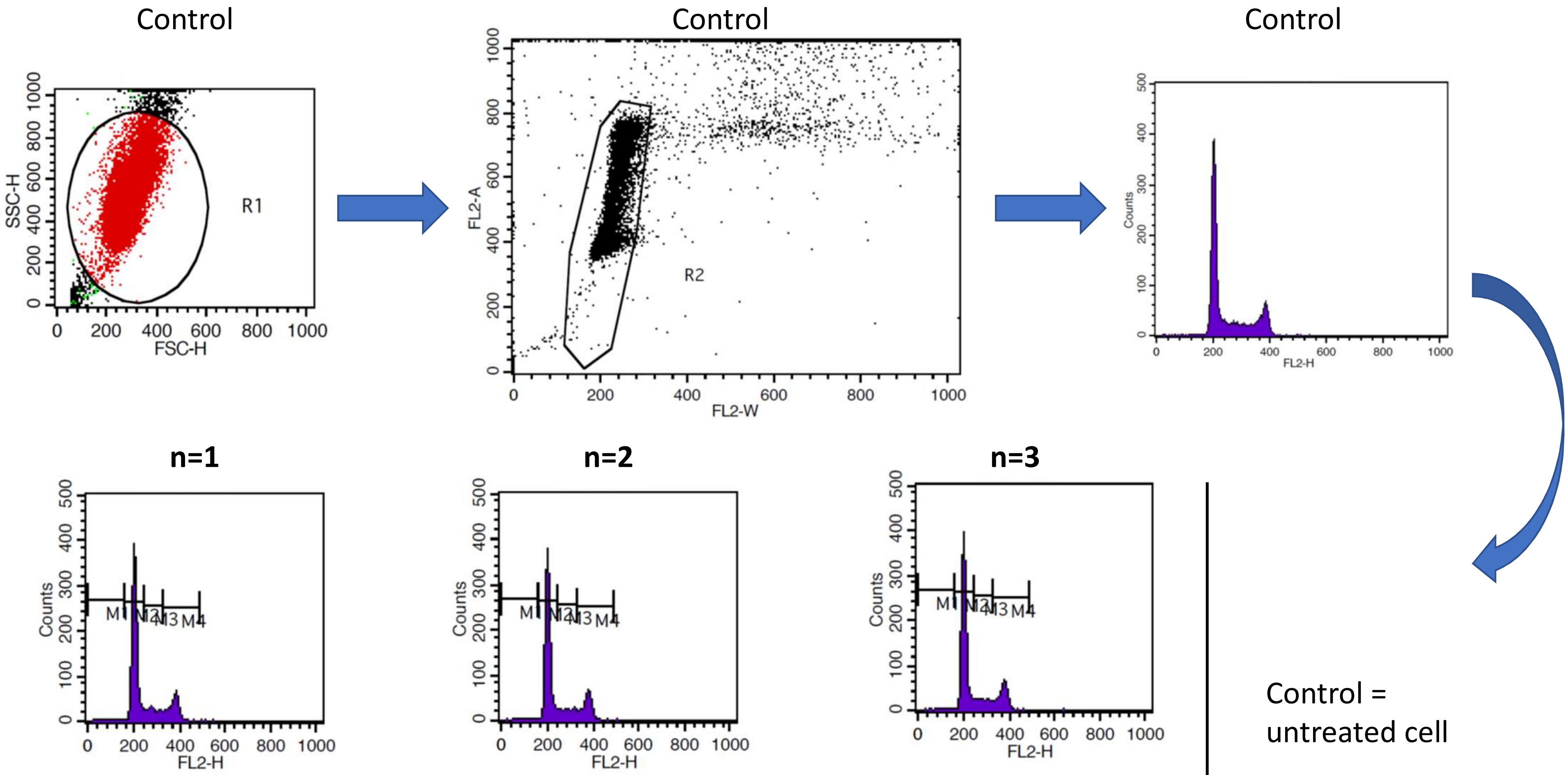

Marker	% Gated
All	100.00
M1	0.36
M2	65.71
M3	14.79
M4	19.39

Marker	% Gated
All	100.00
M1	0.38
M2	66.05
M3	13.90
M4	20.03

Marker	% Gated
All	100.00
M1	0.29
M2	66.12
M3	13.83
M4	20.19

Control = untreated cell

n=1

100 nM $1,25(OH)_2D_3$ 24 h

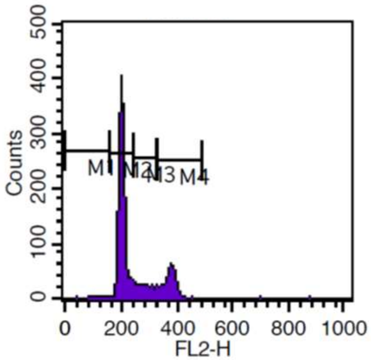

Marker	% Gated
All	100.00
M1	0.20
M2	67.25
M3	14.42
M4	18.47

100 nM Calcipotriol 24 h

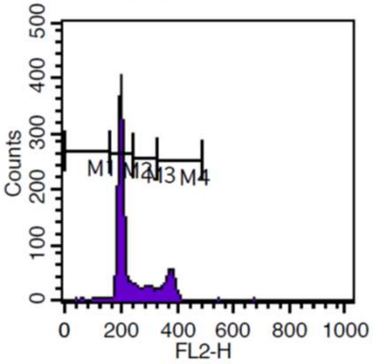

Marker	% Gated
All	100.00
M1	0.20
M2	68.20
M3	14.07
M4	17.88

n=2

100 nM $1,25(OH)_2D_3$ 24 h

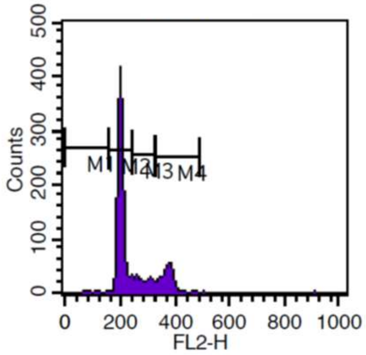

Marker	% Gated
All	100.00
M1	0.16
M2	67.22
M3	14.61
M4	18.31

100 nM Calcipotriol 24 h

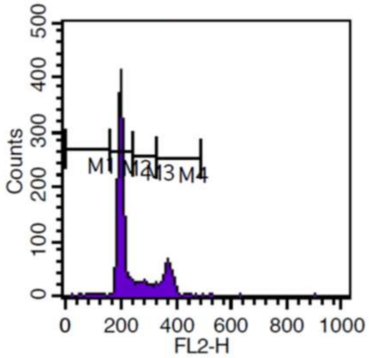

Marker	% Gated
All	100.00
M1	0.26
M2	68.03
M3	13.87
M4	18.27

n=3

100 nM $1,25(OH)_2D_3$ 24 h

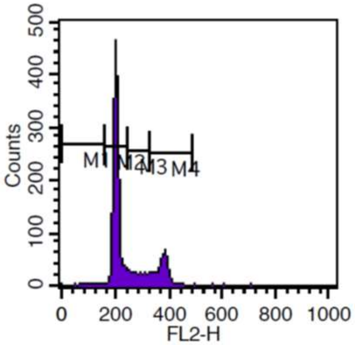

Marker	% Gated
All	100.00
M1	0.27
M2	68.44
M3	14.47
M4	17.15

100 nM Calcipotriol 24 h

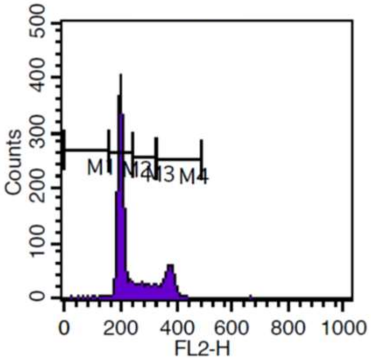

Marker	% Gated
All	100.00
M1	0.23
M2	67.53
M3	14.49
M4	18.16

n=1

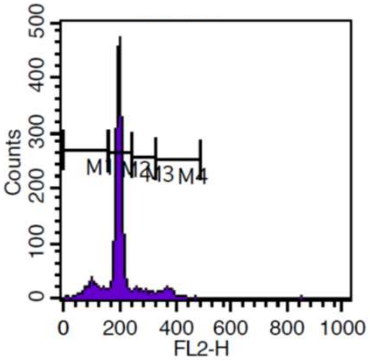

Marker	% Gated
All	100.00
M1	12.86
M2	75.78
M3	6.07
M4	5.55

n=2

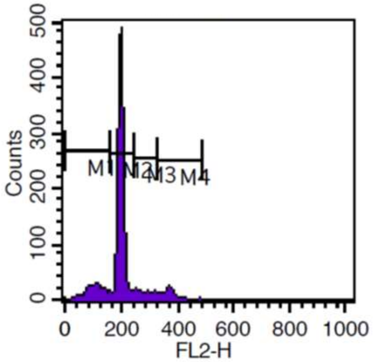

Marker	% Gated
All	100.00
M1	13.97
M2	73.91
M3	6.69
M4	5.68

n=3

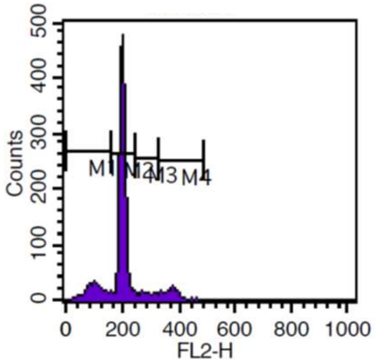

Marker	% Gated
All	100.00
M1	14.24
M2	75.23
M3	5.04
M4	5.68

500 nM Cediranib 72 h

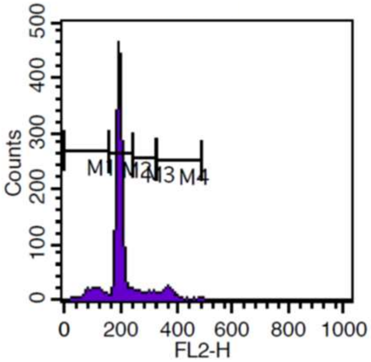

Marker	% Gated
All	100.00
M1	9.05
M2	77.69
M3	6.63
M4	6.81

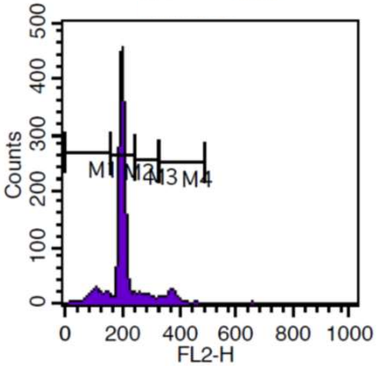

Marker	% Gated
All	100.00
M1	10.41
M2	76.51
M3	6.50
M4	6.75

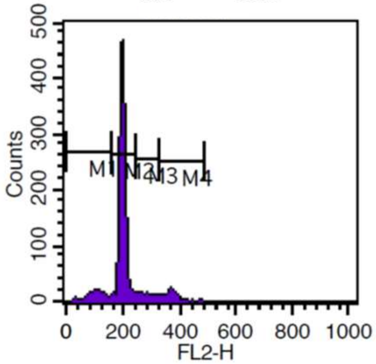

Marker	% Gated
All	100.00
M1	9.37
M2	77.02
M3	7.05
M4	6.78

1000 nM Cediranib 72 h

100 nM $1,25(OH)_2D_3$ 24 h → 500 nM Cediranib 72 h

n=1

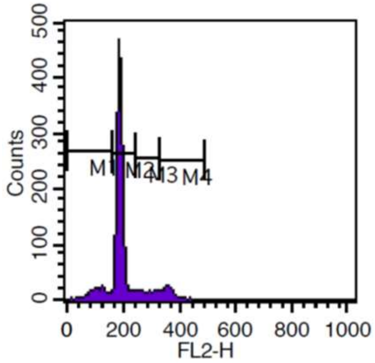

Marker	% Gated
All	100.00
M1	8.11
M2	77.00
M3	8.03
M4	7.22

n=2

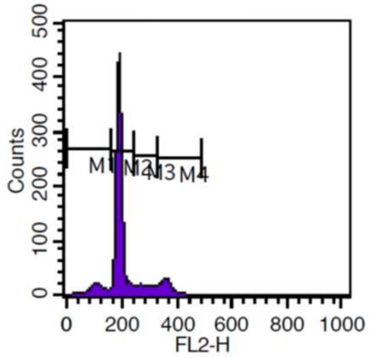

Marker	% Gated
All	100.00
M1	7.65
M2	75.37
M3	8.34
M4	8.89

n=3

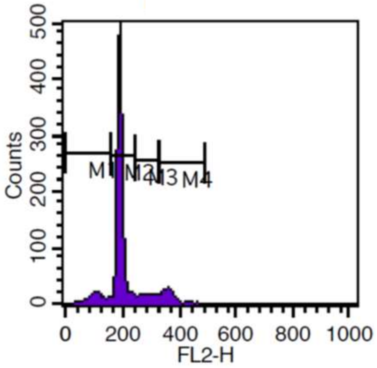

Marker	% Gated
All	100.00
M1	7.92
M2	75.74
M3	7.92
M4	8.61

100 nM $1,25(OH)_2D_3$ 24 h → 1000 nM Cediranib 72 h

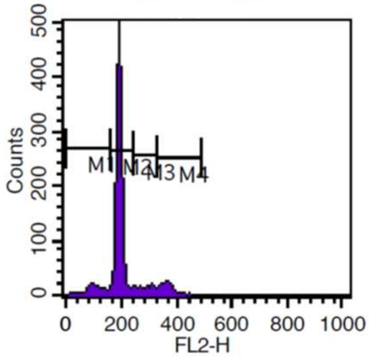

Marker	% Gated
All	100.00
M1	7.21
M2	78.56
M3	6.55
M4	7.94

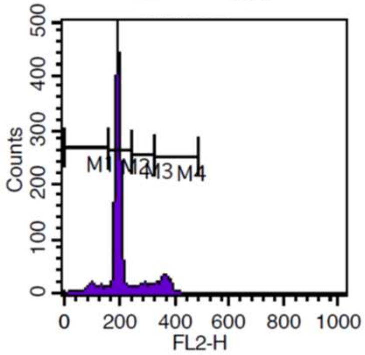

Marker	% Gated
All	100.00
M1	6.86
M2	76.28
M3	7.37
M4	9.76

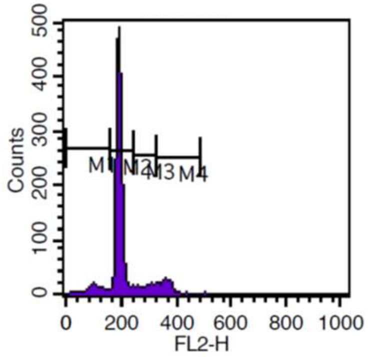

Marker	% Gated
All	100.00
M1	6.49
M2	78.38
M3	7.02
M4	8.33

n=1 **n=2** **n=3**

100 nM Calcipotriol 24 h → 500 nM Cediranib 72 h

n=1

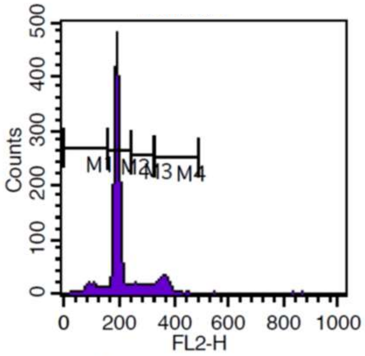

Marker	% Gated
All	100.00
M1	7.12
M2	74.51
M3	8.62
M4	10.01

n=2

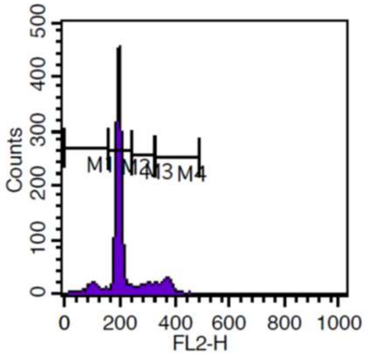

Marker	% Gated
All	100.00
M1	6.25
M2	74.97
M3	8.48
M4	10.53

n=3

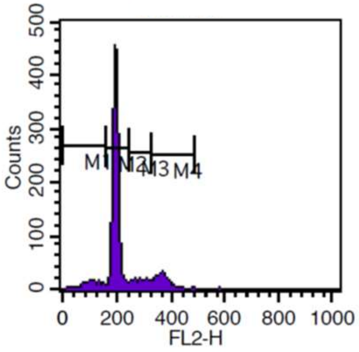

Marker	% Gated
All	100.00
M1	6.06
M2	75.00
M3	8.43
M4	10.74

100 nM Calcipotriol 24 h → 1000 nM Cediranib 72 h

n=1

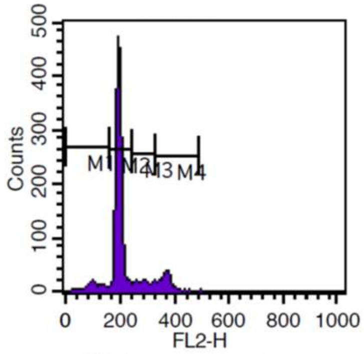

Marker	% Gated
All	100.00
M1	5.99
M2	74.64
M3	8.87
M4	10.75

n=2

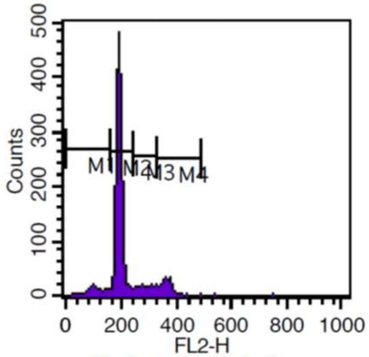

Marker	% Gated
All	100.00
M1	6.38
M2	75.23
M3	8.90
M4	9.65

n=3

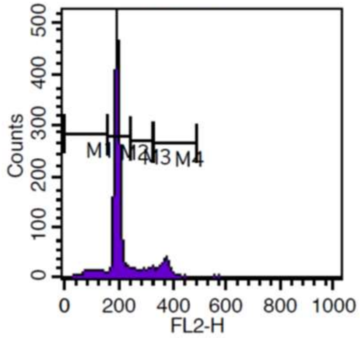

Marker	% Gated
All	100.00
M1	5.87
M2	76.27
M3	8.36
M4	9.72
