# Supplementary material for: Vitamin D Enhances Anticancer Properties of Cediranib, a VEGFR Inhibitor, by Modulation of VEGFR2 Expression in Melanoma Cells
Source: Front Oncol. 2021 Dec 24;11:763895. doi: 10.3389/fonc.2021.763895 (PMC8740239; doi:10.3389/fonc.2021.763895)
Supplement: Supplementary Figure 2 [file Image_2.pdf]

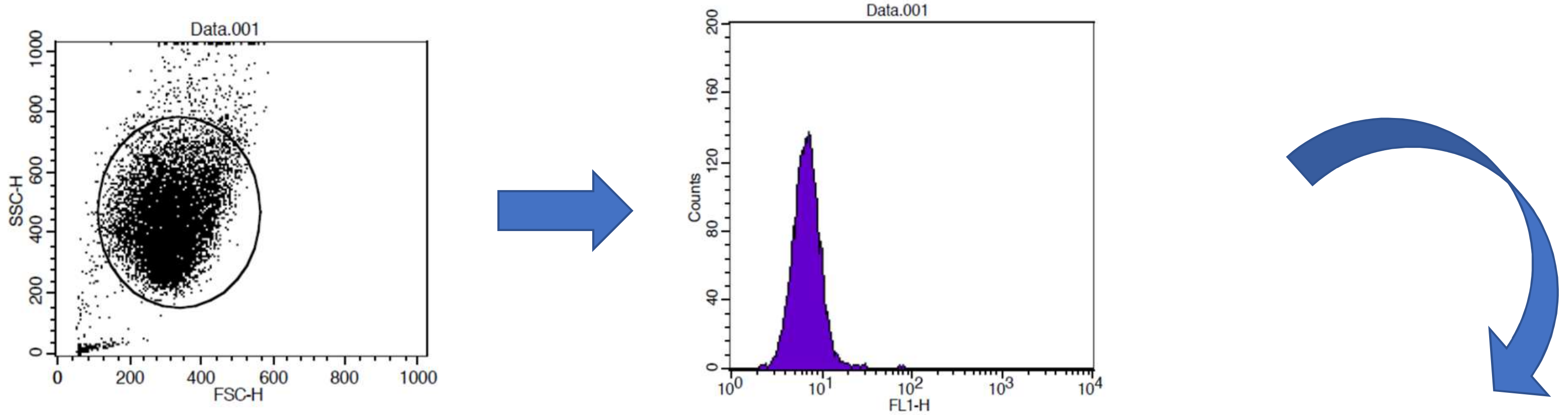

Control = untreated cells, stained with anti-VEGFR2 antibody

Control, unstained cells

n=1

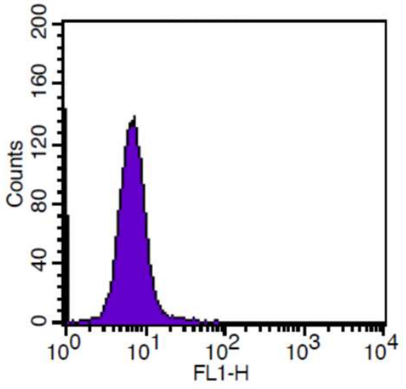

Marker	Mean	Geo Mean
All	7.18	6.64

n=2

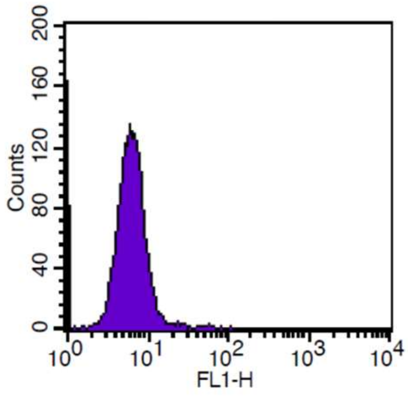

Marker	Mean	Geo Mean
All	6.47	5.96

n=3

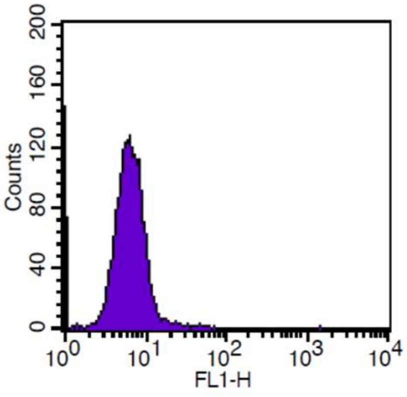

Marker	Mean	Geo Mean
All	6.71	6.09

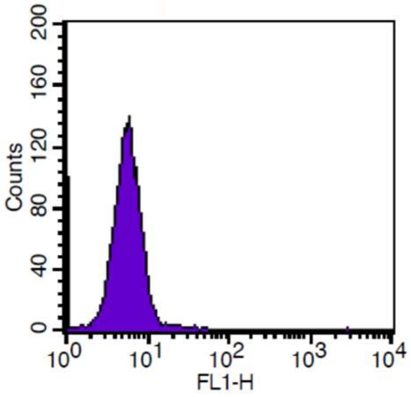

Marker	Mean	Geo Mean
All	6.15	5.44

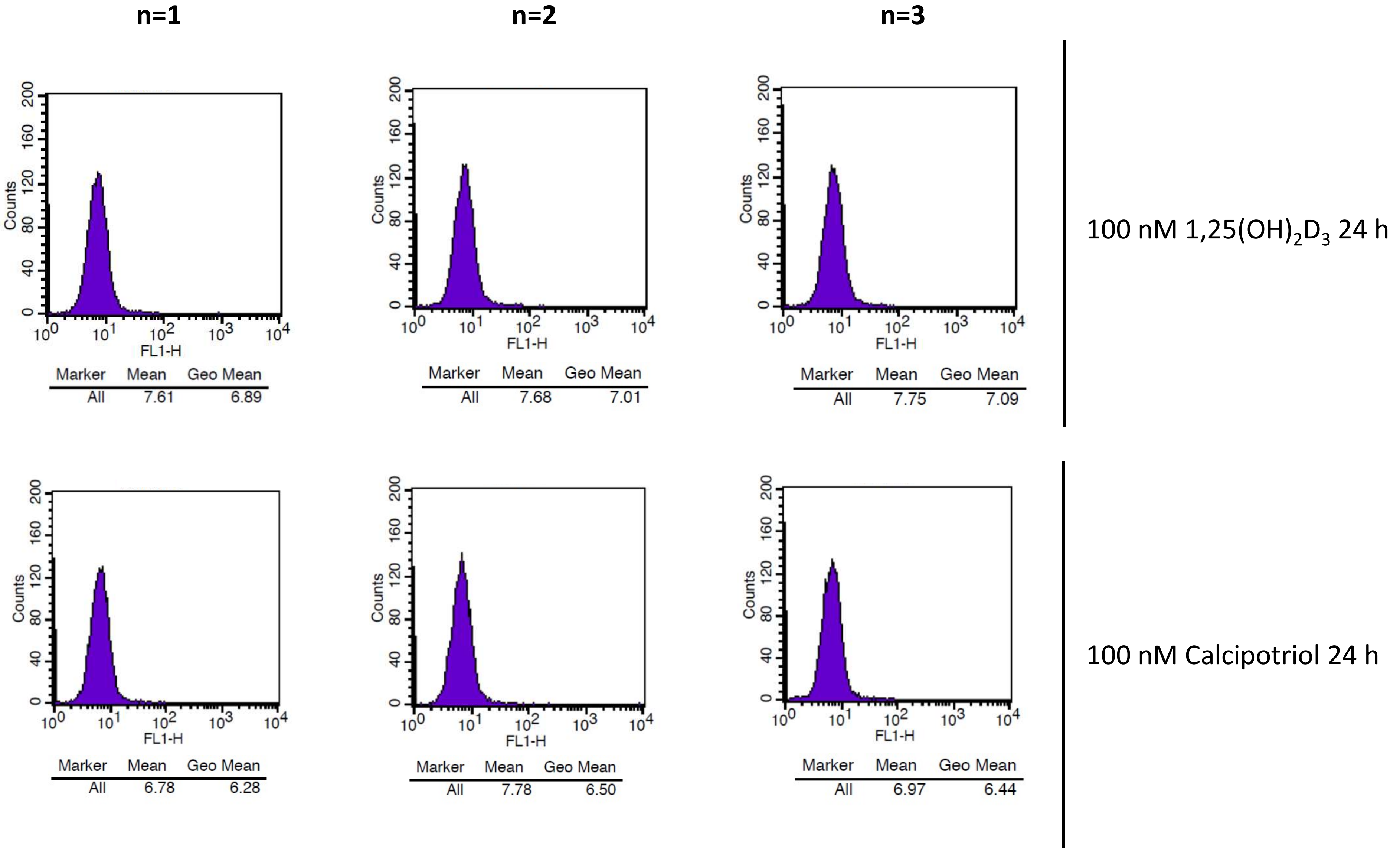
n=1
n=2
n=3
100 nM 1,25(OH)$_2$D$_3$ 24 h
100 nM Calcipotriol 24 h
Counts
FL1-H
Marker Mean Geo Mean
All 7.61 6.89
All 7.68 7.01
All 7.75 7.09
All 6.78 6.28
All 7.78 6.50
All 6.97 6.44

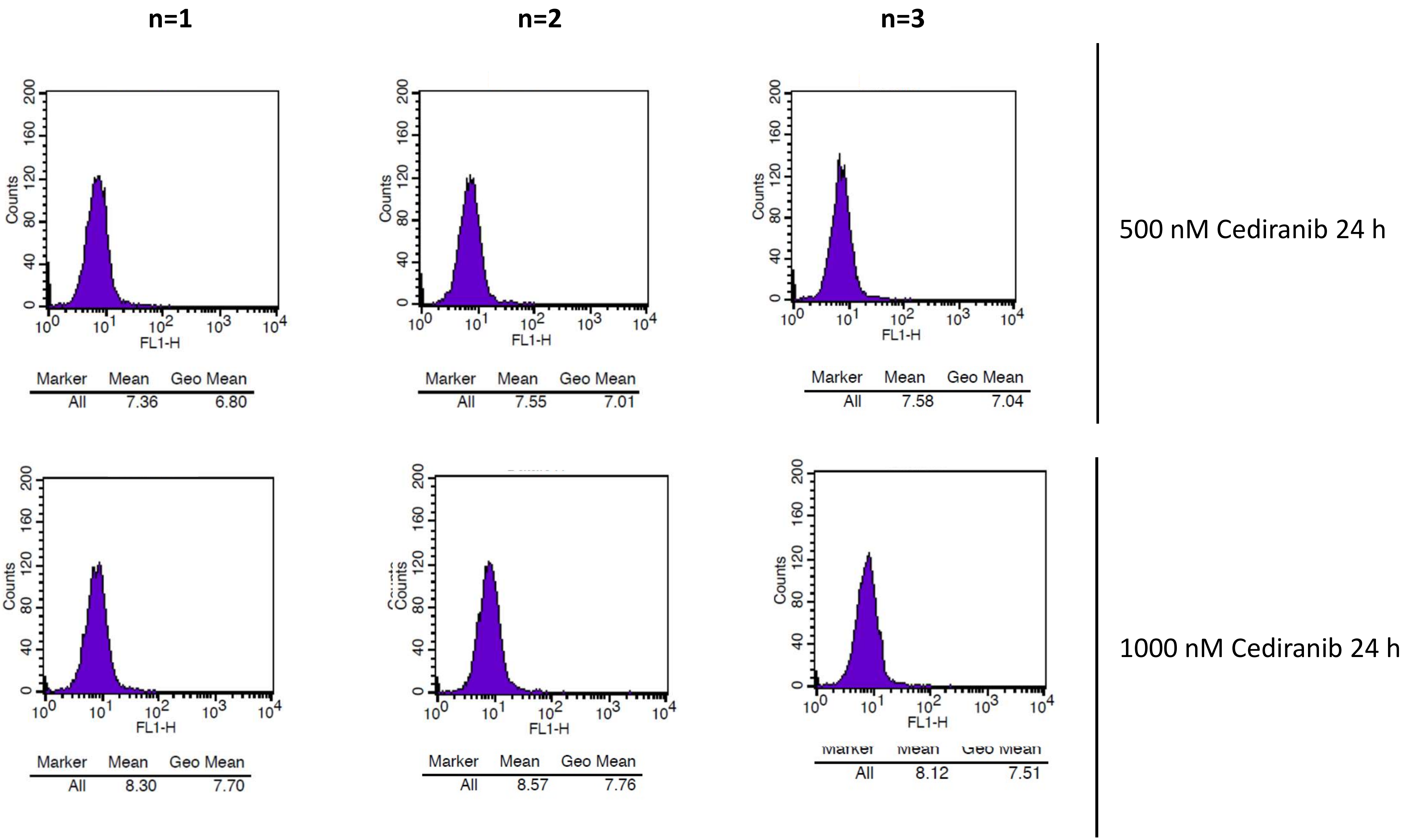

n=1
n=2
n=3
500 nM Cediranib 24 h
1000 nM Cediranib 24 h
Counts
FL1-H
Marker Mean Geo Mean
All 7.36 6.80
All 7.55 7.01
All 7.58 7.04
All 8.30 7.70
All 8.57 7.76
All 8.12 7.51

n=1 n=2 n=3

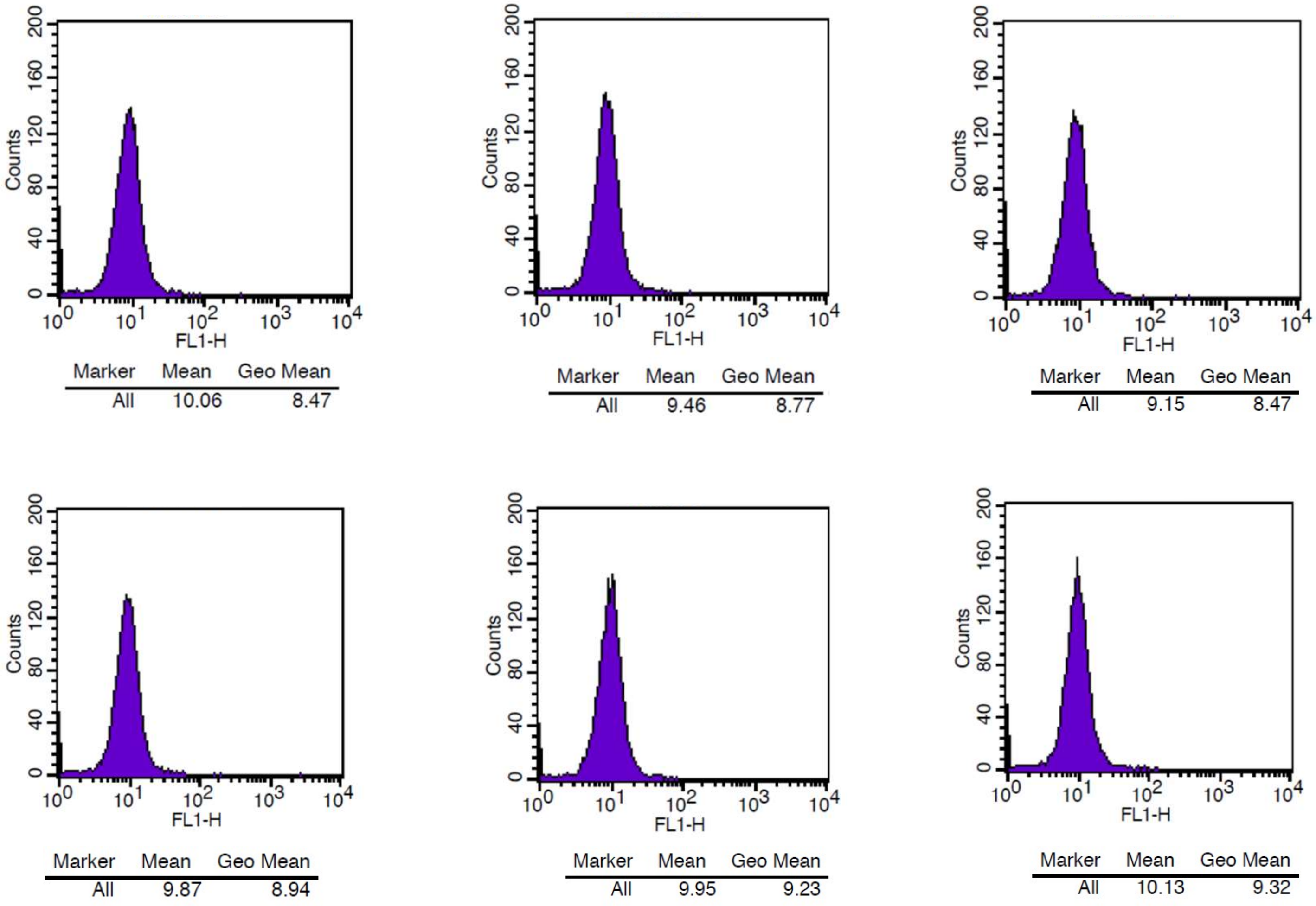

100 nM $1,25(OH)_2D_3$ 24 h → 500 nM Cediranib 24 h

100 nM $1,25(OH)_2D_3$ 24 h → 1000 nM Cediranib 24 h

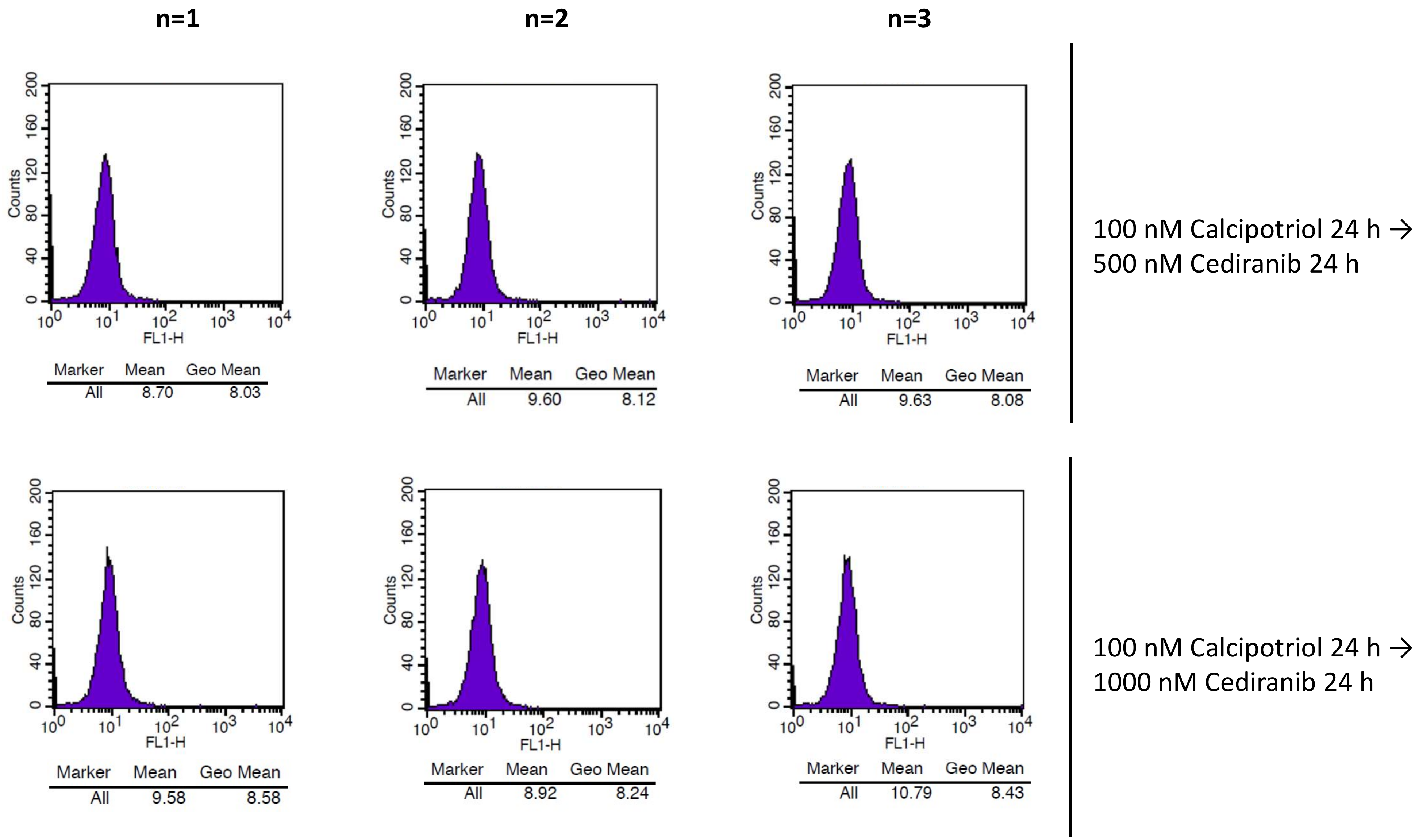

n=1
n=2
n=3
Counts
FL1-H
Marker Mean Geo Mean
All 8.70 8.03
All 9.60 8.12
All 9.63 8.08
100 nM Calcipotriol 24 h →
500 nM Cediranib 24 h
All 9.58 8.58
All 8.92 8.24
All 10.79 8.43
100 nM Calcipotriol 24 h →
1000 nM Cediranib 24 h
